# 4188 Consistent Differences in Lumbar Spine Alignment Between Low Back Pain Subgroups and Sexes during Clinical and Functional Sitting Tests

**DOI:** 10.1017/cts.2020.118

**Published:** 2020-07-29

**Authors:** Quenten L Hooker, Vanessa M. Lanier, Linda R. Van Dillen

**Affiliations:** 1 Washington University in St. Louis

## Abstract

OBJECTIVES/GOALS: Test the validity of a system for subgrouping people with CLBP by comparing lumbar spine alignment in two CLBP subgroups and sexes during clinical tests of maximum flexed and extended sitting and a functional test of preferred sitting. METHODS/STUDY POPULATION: Using the Movement System Impairment classification system, 154 participants with CLBP were subgrouped based on the predominant direction of altered movement and alignment patterns and symptoms during a standardized examination. Participants performed a functional test of preferred sitting followed by clinical tests of maximum flexed and extended sitting in random order. Reflective markers were place superficial to T12, L3 and S1 spinous processes. 3D marker co-ordinate data were collected using an 8 camera motion capture system. Sagittal plane lumbar curvature angle (LCA), defined as the angular distance between T12, L3, and S1 landmarks was calculated for each test. A three-way mixed effect ANOVA model was used to examine the following effects: test, subgroup, sex, test*subgroup, test*sex, subgroup*sex. RESULTS/ANTICIPATED RESULTS: Test: Lumbar alignment patterns were different for flexed [LCA = 7.4° (6.1, 8.7)], extended [LCA = −22.6° (−23.9,−21.3)], and preferred [LCA = −3.8° (−5.2,−2.5)] sitting tests. LBP subgroup: Rotation-extension [LCA = −7.5° (−8.7,−6.3)] had more extended lumbar alignment than rotation [LCA = −5.2° (−6.2,−4.2)]. Sex: Women had more extended lumbar alignment [LCA = −10.3° (−11.2,−9.3)] than men [LCA = −2.5° (−3.7,−1.2)]. Test*sex: The difference in lumbar alignment between women and men was smaller during the flexed sitting test [women = 4.2° (2.5, 5.9), men = 9.9° (7.8, 12.1)], compared to extended [women = −27.5° (−29.2, −25.8), men = −17.0° (−9.2, −14.8)] and preferred [women = −7.4° (−9.1, −5.8), men = −0.3° (−2.5, 1.8)]. The test*subgroup (p = 0.84) and subgroup*sex (p = 0.87) interactions were not significant.

